# Supplementary material for: Comparison of Cancer Patients to Non-Cancer Patients among COVID-19 Inpatients at a National Level
Source: Cancers (Basel). 2021 Mar 21;13(6):1436. doi: 10.3390/cancers13061436 (PMC8004216; doi:10.3390/cancers13061436)
Supplement: Supplementary file 1 [file cancers-13-01436-s001.pdf]

cancers MDPI

Supplementary Material

Table S1. ICD-10 codes used for identification of tumour subtypes.

Tumour subtype	ICD-10 codes	Code wording
Breast	C50	Malignant neoplasm of breast
Colorectal	C18	Malignant neoplasm of colon
	C19	Malignant neoplasm of rectosigmoid junction
	C20	Malignant neoplasm of rectum
	C21	Malignant neoplasm of anus and anal canal
Prostate	C61	Malignant neoplasm of prostate
Lung	C34	Malignant neoplasm of bronchus and lung
Digestive (non-colorectal)	C15-C26 (except C18-C21)	Malignant neoplasms of digestive organs
Urinary tract	C64-C68	Malignant neoplasms of urinary tract
Female genital organs	C51-C58	Malignant neoplasms of female genital organs
Lip, oral cavity and pharynx	C00-C14	Malignant neoplasms of lip, oral cavity and pharynx
Skin	C43-C44	Melanoma and other malignant neoplasms of skin
Hematological	C81-C96	Malignant neoplasms, stated or presumed to be primary, of lymphoid, hematopoietic and related tissue
Other cancers including		
Mesothelial and soft tissue	C45-C49	Malignant neoplasms of mesothelial and soft tissue
Respiratory and intrathoracic organs (not lung)	C30-C39 (except C34)	Malignant neoplasms of respiratory and intrathoracic organs
Bone and articular cartilage	C40-C41	Malignant neoplasms of bone and articular cartilage
Endodrine glands	C73-C75	Malignant neoplasms of thyroid and other endocrine glands
Other male cancers	C60	Malignant neoplasm of penis
	C62	Malignant neoplasm of testis
	C63	Malignant neoplasm of other and unspecified male genital organs
Central nervous system	C69-C72	Malignant neoplasms of eye, brain and other parts of central nervous system

Table S2. Main baseline characteristics of patients hospitalized in France for COVID-19 (from March 1st, 2020, to April 30th, 2020) according to the presence of cancer.

	Cancer	Without cancer	*p*-value
**Number of patients**	6,201	83,329	
**Male gender (n, %)**	3,708 (59·8%)	43,787 (52·56%)	<0·01
**Age**			<0·01
Mean+/-std	72 +/- 13	65 +/- 20	
Med [Q1-Q3]	73 [64-82]	67 [51-81]	
Min-Max	1-100	0-106	
<18	23 (0·4)	1,204 (1·4)	
18-30	46 (0·7)	4,338 (5·2)	
31-40	82 (1·3)	5,983 (7·2)	
41-50	227 (3·7)	8,665 (10·4)	
51-60	694 (11·2)	12,416 (14·9)	
61-70	1,457 (23·5)	13,888 (16·7)	
71-80	1,791 (28·9)	14,474 (17·4)	
81-90	1,534 (24·7)	16,307 (19·6)	
>90	347 (5·6)	6,054 (7·3)	
**Social deprivation score**			
Mean+/-std	-0·44 +/- 1·78	-0·26 +/- 1·78	<0·01
Med [Q1-Q3]	-0,21 [-1,40 - 0,77]	-0,14 [-1,22 - 0,93]	
Lowest	1,634 (27·5)	19,720 (24·9)	<0·01
Second	1,455 (24·5)	19,119 (24·1)	

Third	1,531 (25·8)	20,437 (25·8)	
Highest	1,317 (22·2)	19,969 (25·2)	
**Comorbidities**			
Hypertension	2,216 (35·7)	27,406 (32·9)	<0·01
Dementia	381 (6·1)	6,361 (7·6)	<0.01
HIV	32 (0·5)	400 (0·5)	0·69
Heart failure	581 (9·4)	6,553 (7·9)	<0·01
Chronic respiratory disease	120 (1·9)	1,313 (1·6)	0.03
Chronic kidney disease	620 (10·0)	6,838 (8·2)	<0.01
Cirrhosis	134 (2·2)	584 (0·7)	<0·01
Diabetes	1,209 (19·5)	15,841 (19·0)	0.35
Hypertension	2,192 (36.13)	27,430 (32.86)	<0.01
Peripheral vascular disease	298 (4·8)	2,572 (3·1)	<0·01
Obese or overweight	425 (6·9)	9,691 (11·6)	<0·01
Obese	354 (5·7)	8,257 (9·9)	<0·01
Dyslipidemia	386 (6·2)	4,103 (4·9)	<0·01
Deficiency Anemia	339 (5·5)	3,162 (3·8)	<0·01
BPCO	481 (7·8)	4,385 (5·3)	<0·01
Pulmonary bacterial infection	441 (7·1)	5,845 (7·0)	0·77

Table S3. Percentage of tumour subtypes among cancer patients hospitalized in France for COVID-19 (from March 1st, 2020, to April 30th, 2020) according to age.

	<18	18-30	31-40	41-50	51-60	61-70	71-80	81-90	>90
N	23	42	78	215	652	1328	1657	1403	324
Hematological	47·8	47·6	26·9	24·7	20·3	22·2	24·4	27·4	21
Lung	0	4·8	6·4	13·5	19·3	21·4	16·7	9·4	5·9
Digestive (non-colorectal)	0	9·5	5·1	10·2	12·7	13	12·1	8·1	8·3
Prostate	0	0	0	1·9	3·4	6·8	11·8	17·8	18·5
Breast	0	2·4	25·6	21,9	13·5	8·8	7·2	8·6	14·8
Colorectal	0	7·1	12·8	5·6	7·8	8·8	9	9·4	13·9
Urinary tract	8·7	0	2·6	4·7	5·5	4·7	6·7	8·6	6·2
Other cancers	39·1	26·2	10·3	9·3	6·6	5·6	4·6	3·8	2·8
Female genital organs	4·4	2·4	3·9	3·7	3·7	3·2	3·4	3·1	1·2
Lip, oral cavity and pharynx	0	0	3·9	1·9	6·1	4·5	2·6	0·8	0·3
Skin	0	0	2·6	2·8	1·2	1·1	1·6	3	7·1

Table S4. Complications and main outcomes of patients hospitalized in France for COVID-19 (from March 1st, 2020, to April 30th, 2020) according to the presence of cancer.

	Cancer	Without cancer	*p*-value
**Number of patients**	6,201	83,329	
Acute respiratory failure	1,881 (30·3)	22,436 (26·9)	<0·01
Pulmonary embolism	273 (4·4)	2,813 (3·4)	<0·01
Venous thrombosis (including pulmonary embolism)	379 (6·1)	3,988 (4·8)	<0·01
Septic shock	195 (3·1)	2,356 (2·8)	0.15
Myocardial infarction	27 (0·4)	531 (0·6)	0.05
Atrial fibrillation	974 (15·7)	10,155 (12·2)	<0·01
Stroke	71 (1·1)	997 (1·2)	0·72
Hemorrhagic stroke	19 (0·3)	234 (0·3)	0·71
Ischemic stroke	40 (0·7)	674 (0·8)	0·16
Transient Ischemic Attack	15 (0·2)	146 (0·2)	0·23
Acute kidney failure	503 (8.1)	5,258 (6.3)	<0.01
Intensive care support	930 (15·0)	13,655 (16·4)	<0.01
In-hospital death	2,047 (33·0)	13,057 (15·7)	<0·01

Table S5. Intensive care support and case fatality rates of patients hospitalized in France for COVID-19 (from March 1st, 2020, to April 30th, 2020) according to the presence of cancer and according to age.

Cancer									
**Age strata (years)**	**<18**	**18-30**	**31-40**	**41-50**	**51-60**	**61-70**	**71-80**	**81-90**	**>90**
Number	23	46	82	227	694	1,457	1,791	1,534	347
Intensive care support (%)	21·7 *	26·1 *	19·5 *	15·9	20·9	20·9 $	17·5 $	5·8	2·6
In-hospital death (%)	8·7 *	8·7 *	9·8 *	22·5 *	23·1 *	28·9 *	35·0 *	40·7 *	43·5 *
**Without cancer**									
**Age strata (years)**	**<18**	**18-30**	**31-40**	**41-50**	**51-60**	**61-70**	**71-80**	**81-90**	**>90**
Number	1,204	4,338	5,983	8,665	12,416	13,888	14,474	16,307	6,054
Intensive care support (%)	2·6 *	5·7 *	10·2 *	15·9	23·4	28.9 $	22·2 $	6·6	2·9
In-hospital death (%)	0·6 *	0·4 *	1·3 *	2·2 *	5·1 *	12·1 *	21·6 *	31·6 *	35·9 *

* significantly higher in the cancer group compared to the non-cancer group ($p < 0·05$); $ significantly lower in the cancer group compared to the non-cancer group ($p < 0·05$)

Table S6. Hierarchical logistic regression to study the risk of in-hospital death regarding patients hospitalized in France for COVID-19 (from March 1st, 2020, to April 30th, 2020) with cancer or not.

	Death	Hospital mortality rate	aOR *	p-value	aOR **	p-value
Cancer	2,047	33·0	2·7 [2·5-2·8]	<0·01	2·2 [2·0-2·3]	<0·01
Without cancer	13,057	15·7	ref		ref	
Solid Cancer with metastasis	693	39·0	3·4 [3·1-3·8]	<0·01	3·6 [3·2-4·0]	<0·01
Solid Cancer without metastasis	690	27·0	2·0 [1·8-2·2]	<0·01	1·4 [1·3-1·5]	<0·01
Hematological cancer	470	33·8	2·8 [2·5-3·1]	<0·01	2·2 [2·0-2·5]	<0·01
Without cancer	13,057	15·7	ref		ref	

aOR: adjusted odds ratio; *Hierarchical model using hospitals as the 2nd level, adjusted odds ratio on sex, dementia, heart failure, chronic respiratory disease, cirrhosis, diabetes, deficiency anemia and pulmonary bacterial infection; **Hierarchical model using geographical unit as the 2nd level, adjusted odds ratio on sex, dementia, heart failure, chronic respiratory disease, cirrhosis, diabetes, deficiency anemia and pulmonary bacterial infection

Table 7. Hierarchical Logistic regression to study the risk of hospital death among cancer patients hospitalized in France for COVID-19 (from March 1st, 2020, to April 30th, 2020), using colorectal cancers as the reference group.

	N	Death	Hospital mortality rate	aOR *	p-value	aOR **	p-value
**All cancer**							
Colorectal	518	142	27·4	ref	-	ref	-
Digestive (non-colorectal)	626	233	37·2	1·6 [1·2-2·0]	<0·01	1·6 [1·3-2·1]	<0·01
Breast	561	133	23·7	0·8 [0·6-1·1]	0·16	1·0 [0·8-1·4]	0·76
Prostate	621	188	30·3	1·2 [0·9-1·5]	0·29	0·9 [0·7-1·2]	0·36
Lung	873	359	41·1	1·8 [1·5-2·3]	<0·01	2·0 [1·6-2·6]	<0·01
Urinary tract	363	122	33·6	1·3 [1·0-1·8]	0·049	1·2 [0·9-1·7]	0·15
Female genital organs	185	54	29·2	1·1 [0·8-1·6]	0·64	1·4 [0·9-2·1]	0·07
Lip, oral cavity and pharynx	162	38	23·5	0·8 [0·5-1·2]	0·32	0·9 [0·6-1·4]	0·73
Skin	121	32	26·5	1·0 [0·6-1·5]	0·83	0·8 [0·5-1·3]	0·44
Other cancers***	303	82	27·1	1·0 [0·7-1·3]	0·91	1·2 [0·8-1·6]	0·37
Hematological	1389	470	33·8	1·4 [1·1-1·7]	0·01	1·4 [1·1-1·8]	<0·01
**Solid Cancer with metastasis**							
Colorectal	244	84	34·4	ref	-	ref	-
Digestive (non-colorectal)	244	109	44·7	1·5 [1·1-2·2]	0·02	1·6 [1·1-2·3]	0·02
Breast	241	77	32·0	0·9 [0·6-1·3]	0·56	1·1 [0·8-1·7]	0·52
Prostate	196	74	37·8	1·2 [0·8-1·7]	0·47	0·9 [0·6-1·3]	0·73
Lung	461	215	46·6	1·7 [1·2-2·3]	<0·01	1·7 [1·2-2·4]	<0·01
Urinary tract	128	54	42·2	1·4 [0·9-2·2]	0·14	1·4 [0·9-2·1]	0·15
Female genital organs	119	41	34·5	1·0 [0·6-1·6]	0·99	1·2 [0·7-2·0]	0·44

Lip, oral cavity and pharynx	45	12	26·7	0·7 [0·3-1·4]	0·31	0·7 [0·4-1·5]	0·37
Skin	43	12	27·9	0·7 [0·4-1·5]	0·41	0·7 [0·4-1·5]	0·41
Other cancers***	54	15	27·8	0·7 [0·4-1·4]	0·35	0·8 [0·4-1·6]	0·53
**Solid Cancer without metastasis**							
Colorectal	274	58	21·2	ref	-	ref	-
Digestive (non-colorectal)	382	124	32·5	1·8 [1·2-2·6]	<0·01	2·0 [1·3-3·0]	<0·01
Breast	320	56	17·5	0·8 [0·5-1·2]	0·26	1·1 [0·7-1·7]	0·63
Prostate	425	114	26·8	1·4 [0·9-2·0]	0·09	1·0 [0·7-1·4]	0·93
Lung	412	144	35·0	2·0 [1·4-2·9]	<0·01	2·4 [1·7-3·5]	<0·01
Urinary tract	235	68	28·9	1·5 [1·0-2·3]	0·04	1·3 [0·9-2·0]	0·20
Female genital organs	66	13	19·7	0·9 [0·5-1·8]	0·79	1·4 [0·7-2·9]	0·32
Lip, oral cavity and pharynx	117	26	22·2	1·1 [0·6-1·8]	0·82	1·5 [0·9-2·6]	0·12
Skin	78	20	25·6	1·3 [0·7-2·3]	0·40	1·0 [0·5-1·8]	0·91
Other cancers***	249	67	26·9	1·4 [0·9-2·1]	0·13	2·0 [1·3-3·0]	<0·01

aOR: adjusted odds ratio; *Hierarchical model using hospitals as the 2nd level, adjusted odds ratio on sex, dementia, heart failure, chronic respiratory disease, cirrhosis, diabetes, deficiency anemia and pulmonary bacterial infection; **Hierarchical model using geographical unit as the 2nd level, adjusted odds ratio on sex, dementia, heart failure, chronic respiratory disease, cirrhosis, diabetes, deficiency anemia and pulmonary bacterial infection; *** including mesothelial and soft issue, respiratory and intrathoracic organs (except lung), bone and articular cartilage, endocrine glands, other male cancers and central nervous system

Table S8. Tumour subtypes for cancer patients (with or without metastasis) hospitalized in France for COVID-19 (from March 14th, 2020, to April 30th, 2020).

	**All cancer n=5,348**		Solid Metastatic cancer n=1,624		Solid Cancer without metastasis n=2,413	
	**n**	**%**	n	%	n	%
Hematological	1·311	24·5	-	-	-	-
Lung	794	14·8	404	24·9	390	16·2
Digestive (non-colorectal)	583	10·9	227	14·0	356	14·8
Prostate	587	11·0	183	11·3	404	16·7
Breast	526	9·8	220	13·6	306	12·7
Colorectal	487	9·1	229	14·1	258	10·7
Urinary tract	340	6·4	118	7·3	222	9·2
Other cancers*	283	5·3	49	3·0	234	9·7
Female genital organs	173	3·2	112	6·9	61	2·5
Lip, oral cavity and pharynx	153	2·9	44	2·7	109	4·5
Skin	111	2·1	38	2·3	73	3·0

* including mesothelial and soft issue, respiratory and intrathoracic organs (except lung), bone and articular cartilage, endocrine glands, other male cancers and central nervous system

Table S9. Main baseline characteristics of patients hospitalized in France for COVID-19 (from March 14th, 2020, to April 30th, 2020) according to the presence of cancer (with or without metastasis) and cancer types.

	**Hematological cancer**	**Solid Metastatic cancer**	**Solid Cancer without metastasis**	**Without cancer**	**p-value***
**Number of patients**	1,311	1,624	2,413	79,650	
**Male gender (n, %)**	762 (58·1)**	909 (56·0)**	1,541 (63·9)**	41,867 (52·6)	<0·01
**Age**					<0·01
Mean+/-std	72 +/- 15**	70 +/- 13**	74 +/- 13**	65 +/- 20	
Med [Q1-Q3]	74 [64-83]	71 [62-80]	75 [66-84]	67 [51-81]	
**Social deprivation score**					
Mean+/-std	-0·43 +/- 1·83 [$]	-0·42 +/- 1·72 [$]	-0·44 +/- 1·79 [$]	-0·25 +/- 1·78	<0·01

Med [Q1-Q3]	-0,17 [-1,41 - 0,86]	-0,19 [-1,38 - 0,76] $	-0,21 [-1,39 - 0,77] $	-0,13 [-1,22 - 0,93]	
Lowest (<-1.233)	341 (27·2)**	431 (27·6)**	625 (27·0)**	18,819 (24·8)	<0·01
Second ([-1.23;-0.145[)	301 (24·0)	388 (24·8)	607 (26·3)	18,921 (25·0)	
Third ([-0.145;-0.928[)	328 (26·2)	419 (26·8)	589 (25·5)	19,247 (25·4)	
Highest (≥0.928)	282 (22·5) $	324 (20·7) $	491 (21·2) $	18,818 (24·8)	
**Hospital type admission**					
Public	1,215 (92.7)**	1,364 (84.0) $	2,128 (88.2)	70,844 (88.9)	<0·01
Private	96 (7.3) $	260 (16.0) **	285 (11.8)	8,806 (11.1)	
**Comorbidities**					
Hypertension	483 (36·8)**	461 (28·4)$	952 (39·5)**	26,267 (33·0)	<0·01
Dementia	75 (5·7) $	55 (3·4) $	200 (8·3)	6,101 (7·7)	<0·01
HIV	8 (0·6)	5 (0·3)	15 (0·6)	390 (0·5)	0·37
Heart failure	148 (11·3)**	112 (6·9)	226 (9·4)**	6,155 (7·7)	0·01
Chronic respiratory disease	21 (1·6)	33 (2·0)	46 (1·9)	1,234 (1·6)	0·12
Chronic kidney disease	151 (11·5)**	93 (5·7) $	283 (11·7)**	6,421 (8·1)	<0·01
Cirrhosis	10 (0·8)	22 (1·4)**	87 (3·6)**	554 (0·7)	<0·01
Diabetes	224 (17·1)	272 (16·8) $	549 (22·8)**	15,171 (19·1)	< 0·01
Peripheral vascular disease	43 (3·3)	60 (3·7)	152 (6·3)**	2,430 (3·1)	<0·01
Obese or overweight	125 (9·5) $	65 (4·0) $	194 (8·0) $	9,360 (11·8)	<0·01
Obese	101 (7·7) $	58 (3·6) $	159 (6·6) $	7,971 (10·0)	<0·01
Dyslipidemia	91 (6·9)**	67 (4·1)	164 (6·8)**	3,903 (4·9)	<0·01
Deficiency Anemia	75 (5·7)**	61 (3·8)	143 (5·9)**	2,974 (3·7)	<0·01
COPD	61 (4·7)	104 (6.4)**	229 (9.5)**	4,122 (5.2)	<0·01
Pulmonary bacterial infection	123 (9·4)**	85 (5·2) $	161 (6·7)	5,480 (6·9)	0·03
**Complications**					
Acute respiratory failure	445 (33·9)**	467 (28·8)	708 (29·3)**	21,303 (26·8)	<0·01
Pulmonary embolism	55 (4·2)	79 (4·9)**	97 (4·0)	2,734 (3·4)	<0·01
Venous thrombosis	81 (6·2)**	106 (6·5)**	134 (5·6)	3,859 (4·8)	<0·01
Septic shock	66 (5·0)**	24 (1·5) $	76 (3·2)	2,170 (2·7)	<0·01
Myocardial infarction	10 (0·8)	2 (0·1) $	11 (0·5)	496 (0·6)	0·02
Atrial fibrillation	226 (17·2)**	198 (12·2)	409 (17·0)**	9,649 (12·1)	<0·01
Stroke	11 (0·8)	15 (0·9)	34 (1·4)	936 (1·2)	0·37
Hemorrhagic stroke	5 (0·4)	6 (0·4)	6 (0·3)	226 (0·3)	0·77
Ischemic stroke	5 (0·4)	6 (0·4)	22 (0·9)	630 (0·8)	0·13
Transient Ischemic Attack	1 (0·1)	5 (0·3)	6 (0·3)	134 (0·2)	0·27
Acute kidney failure	142 (10·8)**	90 (5.5)	187 (7.8)*	4,887 (6.1)	<0·01
Intensive care support	320 (24·4)**	137 (8·4) $	332 (13·8) $	12,888 (16·2)	<0·01
In-hospital death	428 (32·7)**	605 (37·3)**	635 (26·3)**	12,251 (15·4)	<0·01

* *p*-value related to the comparison of the three groups (solid metastatic cancer, solid cancer without metastasis and without cancer); ** significantly higher in the cancer group compared to the non-cancer group ($p < 0·05$); $ significantly lower in the cancer group compared to the non-cancer group ($p < 0·05$)

Table S10. Logistic regression to study the risk of transfer to intensive care unit (adjusted odds ratio) regarding patients hospitalized in France for COVID-19 (from March 14th, 2020, to April 30th, 2020) with cancer or not.

	**<40**	**41-50**	**51-80**	**81-90**	**>90**
Cancer	3·8 [2·4-6·1]	1·1 [0·8-1·7]	0·7 [0·6-0·8]	0,8 [0,6-0,9]	0·8 [0·4-1·7]
Without cancer	ref	ref	ref	ref	ref
Solid Cancer with metastasis	1·6 [0·6-4·3]	0·7 [0·3-1·6]	0·4 [0·3-0·4]	0,2 [0,1-0,6]	0·7 [0·1-4·8]
Solid Cancer without metastasis	1·9 [0·7-5·3]	0·6 [0·2-1·4]	0·7 [0·6-0·8]	0,9 [0,6-1,2]	1·1 [0·5-2·5]
Without cancer	ref	ref	ref	ref	ref
Hematological cancer	10·6 [5·5-20·3]	3·3 [1·8-6·2]	1·5 [1·3-1·8]	1·1 [0·7-1·6]	0·6 [0·1-4·3]

Adjusted odds ratio on sex, dementia, heart failure, chronic respiratory disease, cirrhosis, diabetes, deficiency anemia and pulmonary bacterial infection

Table S11. Logistic regression to study the risk of in-hospital death regarding patients hospitalized in France for COVID-19 (from March 14th, 2020, to April 30th, 2020) with cancer or not.

	Death	Hospital mortality rate	OR	p-value	aOR *	p-value
Cancer	1,668	31·2	2·6 [2·4-2·7]	<0·01	2·1 [1·9-2·2]	<0·01
Without cancer	12,251	15·4	ref		ref	
Solid Cancer with metastasis	605	37·3	3·3 [2·9-3·6]	<0·01	3·4 [3·0-3·8]	<0·01
Solid Cancer without metastasis	635	26·3	2·0 [1·8-2·2]	<0·01	1·4 [1·2-1·5]	<0·01
Without cancer	12,251	15·4	ref		ref	
Hematological cancer	428	32·7	2·7 [2·4-3·0]	<0·01	2·1 [1·9-2·4]	<0·01

OR: odds ratio ; aOR: adjusted odds ratio; *Adjusted odds ratio on sex, dementia, heart failure, chronic respiratory disease, cirrhosis, diabetes, deficiency anemia and pulmonary bacterial infection

Table S12. Logistic regression to study the risk of hospital death among cancer patients hospitalized in France for COVID-19 (from March 14th, 2020, to April 30th, 2020), using colorectal cancers as the reference group.

	N	Death	Hospital mortality rate	OR	p-value	aOR *	p-value
**All cancer**							
Colorectal	487	128	26·3	ref	-	ref	-
Digestive (non-colorectal)	583	214	36·7	1·6 [1·3-2·1]	<0·01	1·7 [1·3-2·2]	<0·01
Breast	526	120	22·8	0·8 [0·6-1·1]	0·20	1·0 [0·8-1·4]	0·79
Prostate	587	177	30·2	1·2 [0·9-1·6]	0·16	0·9 [0·7-1·2]	0·56
Lung	794	307	38·7	1·8 [1·4-2·3]	<0·01	1·9 [1·5-2·5]	<0·01
Urinary tract	340	110	32·4	1·3 [1·0-1·8]	0·06	1·2 [0·9-1·7]	0·19
Female genital organs	173	47	27·2	1·0 [0·7-1·5]	0·82	1·3 [0·9-2·0]	0·16
Lip, oral cavity and pharynx	153	35	22·9	0·8 [0·5-1·3]	0·40	1·0 [0·6-1·5]	0·90
Skin	111	27	24·3	0·9 [0·6-1·5]	0·67	0·8 [0·5-1·3]	0·32
Other cancers**	283	75	26·5	1·0 [0·7-1·4]	0·95	1·2 [0·9-1·7]	0·30
Hematological	1311	428	32·7	1·4 [1·1-1·7]	0·01	1·4 [1·1-1·8]	0·01
**Solid Cancer with metastasis**							
Colorectal	229	75	32·8	ref	-	ref	-
Digestive (non-colorectal)	227	100	44·1	1·6 [1·1-2·4]	0·01	1·7 [1·1-2·5]	0·01
Breast	220	67	30·5	0·9 [0·6-1·3]	0·60	1·0 [0·7-1·5]	0·90
Prostate	183	70	38·3	1·3 [0·8-1·9]	0·25	1·1 [0·8-1·7]	0·55
Lung	404	173	42·8	1·5 [1·1-2·2]	0·01	1·6 [1·1-2·2]	0·01
Urinary tract	118	48	40·7	1·4 [0·9-2·2]	0·14	1·4 [0·9-2·2]	0·15
Female genital organs	112	37	33·0	1·0 [0·6-1·6]	0·96	1·0 [0·6-1·6]	0·97
Lip, oral cavity and pharynx	44	11	25·0	0·7 [0·3-1·4]	0·31	0·7 [0·3-1·5]	0·39
Skin	38	11	29·0	0·8 [0·4-1·8]	0·64	0·9 [0·4-1·9]	0·73
Other cancers**	49	13	26·5	0·7 [0·4-1·5]	0·40	0·9 [0·4-1·8]	0·71
**Solid Cancer without metastasis**							
Colorectal	258	53	20·5	ref	-	ref	-
Digestive (non-colorectal)	356	114	32·0	1·8 [1·3-2·7]	<0·01	2·0 [1·4-3·0]	<0·01
Breast	306	53	17·3	0·8 [0·5-1·2]	0·33	1·1 [0·7-1·8]	0·61
Prostate	404	107	26·5	1·4 [0·9-2·0]	0·08	1·0 [0·7-1·5]	0·95
Lung	390	134	34·4	2·0 [1·4-2·9]	<0·01	2·4 [1·6-3·6]	<0·01
Urinary tract	222	62	27·9	1·5 [1·0-2·3]	0·06	1·3 [0·9-2·0]	0·22
Female genital organs	61	10	16·4	0·8 [0·4-1·6]	0·47	1·2 [0·5-2·6]	0·67
Lip, oral cavity and pharynx	109	24	22·0	1·1 [0·6-1·9]	0·75	1·5 [0·9-2·7]	0·13
Skin	73	16	21·9	1·1 [0·6-2·0]	0·80	0·8 [0·4-1·6]	0·56
Other cancers**	234	62	26·5	1·4 [0·9-2·1]	0·12	2·0 [1·3-3·1]	<0·01

OR: odds ratio ; aOR: adjusted odds ratio; * adjusted on age, sex, dementia, heart failure, chronic respiratory disease, cirrhosis, diabetes, deficiency anemia and pulmonary bacterial infection; ** including mesothelial and soft issue,

respiratory and intrathoracic organs (except lung), bone and articular cartilage, endocrine glands, other male cancers and central nervous system.

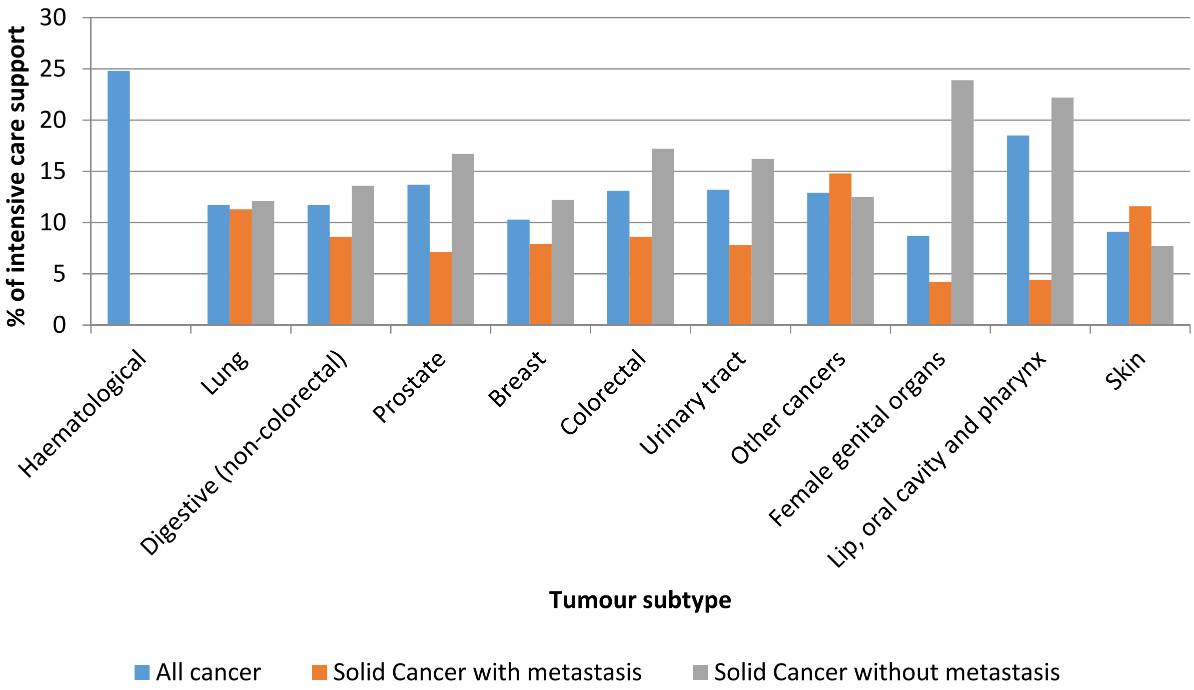

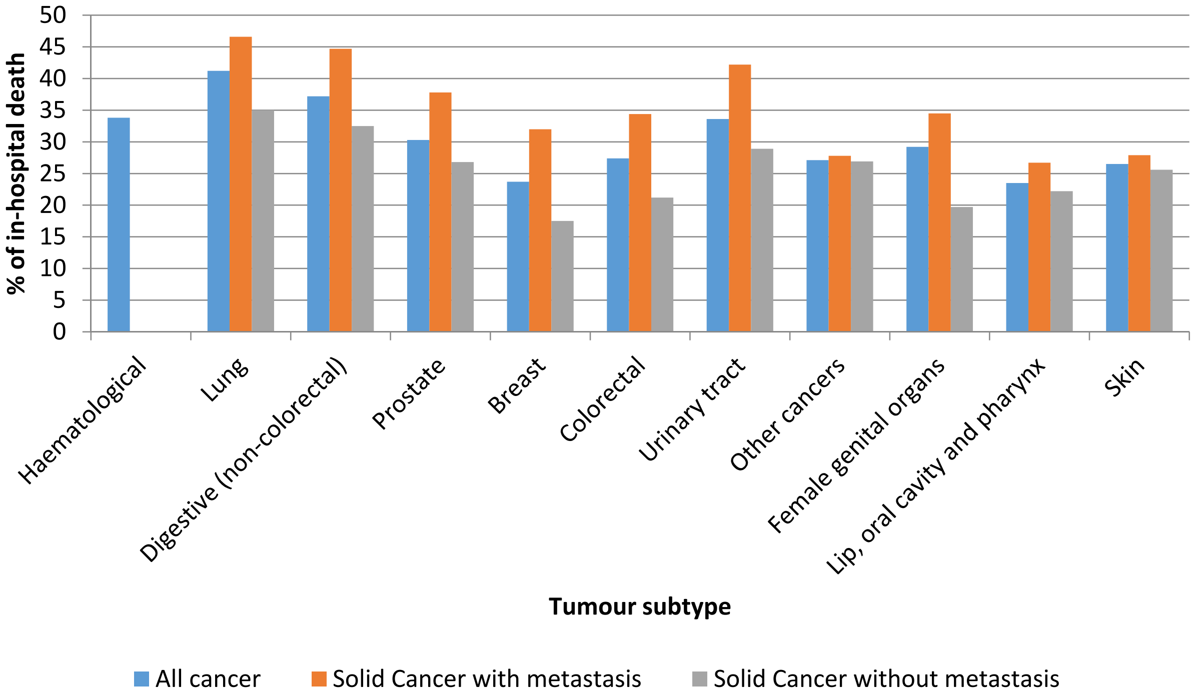

Figure S1. Intensive case support and case fatality rates of cancer patients (with or without metastasis) hospitalized in France for COVID-19 (from March 1st, 2020, to April 30th, 2020) according to tumour subtypes.
